# Anti-tuberculosis drug development *via* targeting the cell envelope of *Mycobacterium tuberculosis*

**DOI:** 10.3389/fmicb.2022.1056608

**Published:** 2022-12-21

**Authors:** Xinyue Xu, Baoyu Dong, Lijun Peng, Chao Gao, Zhiqun He, Chuan Wang, Jumei Zeng

**Affiliations:** ^1^West China-PUMC CC Chen Institute of Health, West China School of Public Health and West China Fourth Hospital, Sichuan University, Chengdu, China; ^2^State Key Laboratory of Biotherapy, Sichuan University, Chengdu, China; ^3^Laboratory of Human Diseases and Immunotherapies, West China Hospital, Sichuan University, Chengdu, China

**Keywords:** *Mycobacterium tuberculosis*, anti-tuberculosis drug, cell envelope, drug target, lead compounds

## Abstract

*Mycobacterium tuberculosis* possesses a dynamic cell envelope, which consists of a peptidoglycan layer, a mycolic acid layer, and an arabinogalactan polysaccharide. This envelope possesses a highly complex and unique structure representing a barrier that protects and assists the growth of *M*. *tuberculosis* and allows its adaptation to the host. It regulates the immune response of the host cells, causing their damage. Therefore, the cell envelope of *M*. *tuberculosis* is an attractive target for vaccine and drug development. The emergence of multidrug-resistant as well as extensively drug resistant tuberculosis and co-infection with HIV prevented an effective control of this disease. Thus, the discovery and development of new drugs is a major keystone for TB treatment and control. This review mainly summarizes the development of drug enzymes involved in the biosynthesis of the cell wall in *M*. *tuberculosis*, and other potential drug targets in this pathway, to provide more effective strategies for the development of new drugs.

## Introduction

Tuberculosis (TB) is an infectious disease caused by *M*. *tuberculosis*. In 2020, approximately 9.9 million people fell ill with TB globally (World Health, 2021). Since early 2020, the COVID-19 pandemic has had a dramatic impact on the outbreak and treatment of TB (Tadolini et al., 2020). The current standard treatment for drug-sensitive TB is a combination of four first-line drugs for 6 months. However, long-term use of multiple drugs may cause adverse drug reactions, which may lead to the interruption of anti-TB treatment, and even develop into multidrug-resistant TB (MDR-TB) and extensively drug-resistant TB (XDR-TB; Miotto et al., 2018). The emergence of multidrug-resistant TB and co-infection with HIV prevented an effective control of the disease (Bell and Noursadeghi, 2018). Therefore, the exploration of new drug targets and new anti-TB compounds is urgently needed.

**Figure 1:**
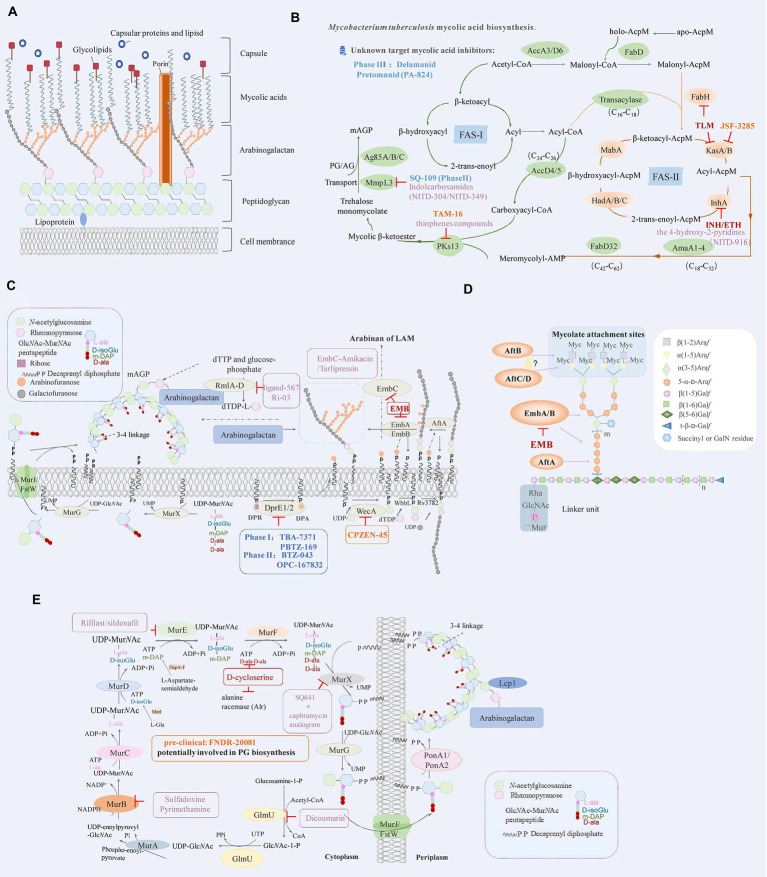
**(A)** Schematic representation of *Mycobacterium tuberculosis* cell envelope. **(B)** *Mycobacterium tuberculosis* mycolic acid biosynthesis. The key enzymes and drug targets of mycolic acids biosynthesis are shown as indicated. FAS-I: fatty acid synthase types I, FAS-II: fatty acid synthase types I. **(C)** Drugs targeting the decaprenylphosphoryl arabinose biosynthesis pathway. DPA, the precursor of arabinan, is synthesized by the epimerization of DPR by DprE1 and DprE2. The key enzymes and drug targets of decaprenylphosphoryl arabinose biosynthesis are shown as indicated. **(D)** Structure of mycobacterial arabinogalactan and roles of key enzymes that are responsible for its biosynthesis. **(E)** The biosynthesis pathway of peptidoglycan in *M*. *tuberculosis* and roles of key enzymes that are responsible for its biosynthesis. The key enzymes and drug targets of peptidoglycan biosynthesis are shown as indicated. The diagram represents the drugs (red) that are effective against *M*. *tuberculosis*, drugs in clinical trials (blue), pre-clinical (orange), and compounds in the discovery stage with promising development (purple).

The structure of the cell envelope of *M*. *tuberculosis* is very different from that of Gram-negative and Gram-positive bacteria. The mycolic acids (MA) of *M*. *tuberculosis* is covalently attached to the peptidoglycan (PG) layer and arabinogalactan (AG) polysaccharide，the whole complex is called mAGP (Kalscheuer et al., 2019). Mycobacterial outer membrane consists of two parts: the innermost leaflet contained mainly the mycolic acids, the outermost leaflet is composed of various glycolipids, trehalose monomycolate (TMM), trehalose 6,6′-dimycolate (TDM), phospholipids, porins, and waxes. (Alderwick et al., 2007; Jankute et al., 2015; Figure 1A). The lipids represents approximately 60% of the dry weight of the cell wall, and the virulence increase when the lipid contents (non-covalently exposed glycolipids, sulfoglycolipids and some phospholipids) increase; thus, the lipid content is closely related to the virulence of the bacteria. This unique cell wall enables the survival of *M*. *tuberculosis* in extremely harsh environments, as well as its survival under the treatment with chemotherapeutic agents (Daffe, 2015; Bhat et al., 2017). Changing the compounds entering pathway and/or increasing its entering amount are the challenges to developing inhibitors targeting the intracellular synthesis stage of bacterial cell wall. With the comprehensive understanding of the key enzymes and extensive studies in cell wall synthesis, the prospect of cell wall as a drug target is quite brilliant. Therefore, the biosynthesis pathways of the precursors of *M*. *tuberculosis* cell wall and their subsequent process of transport and polymerization are effective targets for anti-TB drug development. In this mini review, we summarized the potential drugs and drug targets existing in the biosynthetic pathways of mycobacteria cell envelope.

## Potential drug targets of mycolic acids

Mycolic acids (MA) are important components of the cell wall in *M*. *tuberculosis* (Asselineau and Lederer, 1950); its biosynthetic pathway mainly involves FAS-I (fatty acid synthase type I) and FAS-II (fatty acid synthase type II; Figure 1B). The FAS-I utilizes acetyl-CoA and malonyl-CoA to generate a butyryl-S-enzyme complex; then the butyryl group receives two carbon units from malonyl-CoA. Each round of elongation lengthens the chain by two carbons and finally synthesizes 16- or 18-carbon-containing fatty acids (Jankute et al., 2015). The FAS-II uses ACP transacylase (FabD, Rv2243; Kremer et al., 2001), β-ketoacyl-ACP synthesis III (FabH, Rv0533c; Lai and Cronan, 2003), β-ketoacyl-ACP reductase (MabA/fabG1, Rv1483; Parish et al., 2007), β-hydroxy acyl-ACP dehydratase (HadABC, Rv0635/Rv0636/Rv0637; Sacco et al., 2007; Slama et al., 2016; Farjallah et al., 2021), 2-trans-enoyl-ACP reductase (InhA, Rv1484; Prasad et al., 2021) and β-ketoacyl-ACP synthetase (KasA/B, Rv2245/ Rv2246; Lee and Engels, 2013; Vilcheze et al., 2014) for the extension of the substrate. The acyl-CoA and meroacyl-ACP are synthesized after several cycles. Meromycolyl-AMP and 2-carboxyl-acyl-CoA are generated with the catalysis of FabD32 (Rv3801c) ligase (Portevin et al., 2005; Gavalda et al., 2009) and AccD4/AccD5 (Rv3799c/Rv3280) enzymes (Portevin et al., 2005; Oh et al., 2006). The condensation of meromycolate and C-26 is manipulated by polyketide synthase 13 (Pks13, Rv3800c; Figure 1B; Portevin et al., 2004; Gavalda et al., 2009). Mycolic acids biosynthesis in *M*. *tuberculosis* occurs through the concerted action of more than 20 enzymes that are components of different multi-enzyme complexes. Therefore, this pathway represents an important reservoir of novel targets for the development of new drugs to cure TB, especially in the context of the emergence of drug resistance.

### 2-trans-enoyl-ACP reductase

Isoniazid (INH), with the enoyl-AcpM reductase InhA being the primary target, can attack many targets in *M*. *tuberculosis* after oxidation by KatG (Rv1908c) (catalase-peroxidase). The isonicotinic acyl–NADH complex is created, which blocks the metabolic pathway of mycolic acids in the cell wall by inhibiting 2-trans-enoyl-ACP reductase (InhA) active site, and finally leads *M*. *tuberculosis* death (Vilcheze and Jacobs Jr., 2019). Ethionamide (ETH) is similar in structure to INH and is also a prodrug. It is activated by the enzyme ethA (Rv3854c, a monooxygenase), and binds NAD^+^ to form an ETH-NAD adduct which inhibits the same target site as INH (Vilcheze and Jacobs Jr., 2014).

Aside adduct-forming compounds, there are many inhibitors that directly bind to InhA occupying the fatty-acyl binding site and even bi-substrate inhibitors, that bind simultaneously in both FA and cofactor-binding sites. These directly binding small molecule inhibitors include, arylamides (He et al., 2007), triclosan, diphenyl ethers derivatives (Freundlich et al., 2009; Kamsri et al., 2014), pyrrolidine carboxamide analogs (Kouassi et al., 2015), imidazopiperidine (Wall et al., 2007), and 4-hydroxy-2-pyridones (Figure 1B; Manjunatha et al., 2015; Guardia et al., 2016). The isoniazid-NAD truncated adducts are bi-substrate inhibitors of InhA, the hydrophobic substituents of isoniazid-NAD truncated adducts would be recognized by the fatty-acyl binding site of InhA, and the nicotinamide moiety interacts with the cofactor capsule (Delaine et al., 2010). These inhibitors directly bind to the InhA target without prior activation, avoiding the issues due to clinical drug resistance related to KatG (Prasad et al., 2021). A drug directly targeting InhA in a different location than the activated INH and not requiring activation by KatG may have bactericidal and sterilizing properties superior to those of INH. Since most INH resistance is mediated by mutations in katG, there should also be little or no cross-resistance between a direct inhibitor of InhA and INH. However, these compounds have some limitations. The triclosan, which has been shown associated with the low oral bioavailability (Vosatka et al., 2018), the neurodevelopment impairment (Alfhili et al., 2021), gestational diabetes (Ouyang et al., 2018) and the decline of reproductive function in male mice (Priyanka et al., 2020). Future novel agent should be orally administered, well-tolerated, and with a low propensity to generate resistance.

### β-ketoacyl-ACP synthase (KasA, KasB, and FabH)

The β-ketoacyl-AcpM synthase (KasA) of *M*. *tuberculosis* is an essential enzyme in the mycobacterial fatty acid biosynthesis (FAS-II) pathway and is a potentially promising target for antibacterial drug development. Inhibitors of KasA have been previously reported, and among them, the most significant inhibitor is thiolactomycin (TLM; Figure 1B), which can inhibit KasA, KasB, and FabH (Choi et al., 2000; Kremer et al., 2000). Many *M*. *tuberculosis* beta-ketoacyl-ACP synthase KasA inhibitors lack sufficient potency and/or pharmacokinetic properties. JSF-3285 (Table 1) is an indazole targeting KasA, as demonstrated by the article published in 2020 by Rutgers University (Inoyama et al., 2020). JSF-3285 is a promising preclinical candidate for TB. This study plans to identify and develop novel, small molecule inhibitors of KasA that can be used in combination with other agents to improve the therapeutic effect and to treat drug-resistant forms of TB.

**Table 1:** Molecules in various stages of drug discovery, their targets, chemical structure, efficacy against *Mycobacterium tuberculosis* and some other information.

Target	Development phase	Drug	Chemical structure	Chemical Class	Compounds activity
KasA	Pre-Clinical(Non-GLP)	JSF-3285	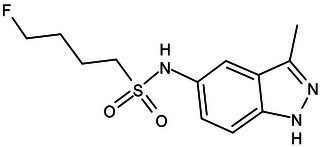	Indazole	MIC of H37Rv: 0.2 μM; hERG inhibition (IC_50_ > 50 μM); Vero CC_50_:170 μM; C_lung_/C_plasma_ at 5 h: 0.75 (Inoyama et al., 2020)
WecA	Pre-Clinical(Non-GLP)	CPZEN-45	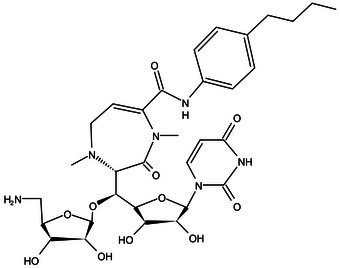	Caprazene nucleoside	MIC of H37Rv: 1.56 μg/mL; MIC of MDR-Mtb: 6.25 μg/mL; poor solubility and low bioavailability (Ishizaki et al., 2013; Salomon et al., 2013)
Pks-13	Lead Optimization	TAM-16	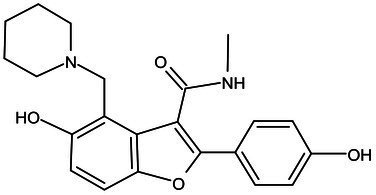	Benzofuran	MIC of Mtb include MDR/XDR-Mtb: (0.05–0.42 μM); has excellent pharmaco-logical and safety profiles; Oral bioavailability (F): 28%; CL_int_: Mouse <0.5, Human <0.5 ml/min/g liver (Aggarwal et al., 2017)
peptidoglycan biosynthesis	Pre-Clinical(GLP)	FNDR-20081	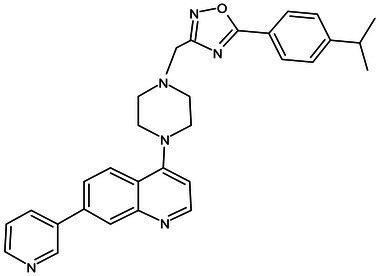	Oxadiazole-piperazine-quinoline	No toxicity to THP-1 and HepG2 cells, (cytotoxicity >64 μg/mL); CYP 3A4 inhibition: IC_50_>25 μM; unstable moderate in HLM and poor in MLM (Kaur et al., 2021)
DprE1	Phase I	PBTZ-169	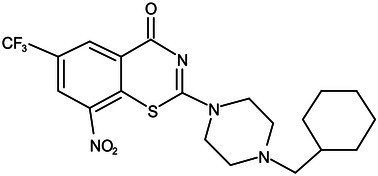	Benzothiazinones	MIC<0.19–0.3 μg/mL; lower cytotoxi-city and better efficacy; The Phase II clinical trial of PBTZ-169 was terminated very slow enrollment (Makarov et al., 2014)
		TBA-7371	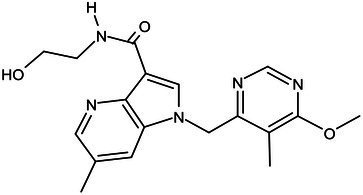	Azaindole	MIC of Mtb: 0.78–3.12 μM; inhibits DprE1 with an IC50 value of 10 nM; Recruiting for Phase II (Shirude et al., 2013; Chatterji et al., 2014)
	Phase II	BTZ-043	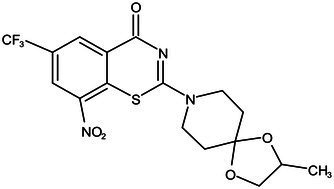	Benzothiazinones	MIC of Mtb: 1 μg/mL; more cytotoxic than INH; Recruiting for Phase III clinical trials (Makarov et al., 2009)
		OPC-167832	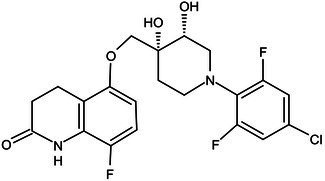	3,4-dihydrocarbostyril derivative	MIC of Mtb, MDR/XDR-Mtb: 0.24-2 μg/mL; Phase Ib/IIa clinical trials to evaluate its safety and efficacy in TB patients (Hariguchi et al., 2020)
MmpL3	Lead Optimization	NITD-304/	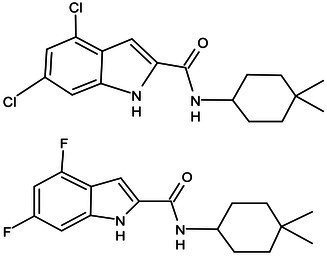	Indolcarboxamides	MIC of H37Rv: 0.008 μg/mL and 0.016 μg/mL; good oral bioavailability (53 and 37%); CC_50_: HepG2>20 μM, THP-1>20 μM; hERG binding and patch clamp IC_50_ were > 30 μM (a low risk of cardiotoxicity; Rao et al., 2013; Li et al., 2017)
		NITD-349			
	PhaseII	SQ-109	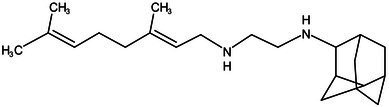	1,2-ethylenediamine	MIC of Mtb include MDR/XDR-Mtb:0.20–0.78 μg/mL; See the text section for other specific information (Sacksteder et al., 2012)
Mycolic acids biosynthesis	Phase III	Delamanid	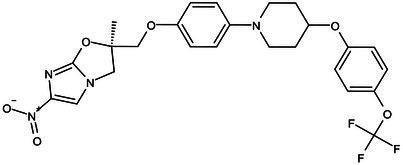	Nitrodihydro imidazooxazole	MIC of Mtb: 0.006–0.024 μg/mL; it does not affect the activity of liver drug enzymes; a combination regimen of delamanid, linezolid, levofloxacin, and pyrazinamide for the treatment of fluoroquinolone-sensitive MDR-TB (Matsumoto et al., 2006; Xavier and Lakshmanan, 2014)
		Pretomanid (PA-824)	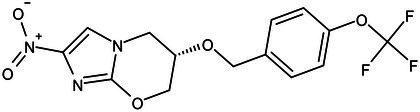	Bicyclic nitroimidazole	MIC of Mtb: 0.031–0.531 μg/mL; combination regimens (BPaL) and (BPaMZ) more effective against drug-resistant TB (Lenaerts et al., 2005; Singh et al., 2008; Burki, 2019; Xu et al., 2019)

### Enzymes related to the last step of mycolic acids biosynthesis

Polyketide synthase 13 (PKs13) is an essential enzyme that forms mycolic acids, and it is required for the formation of the cell wall of *M*. *tuberculosis*. PKs13 is involved in the last step of the mycolic acids biosynthesis pathway and has been widely studied as a drug target for TB (Figure 1B; Yu, M. et al., 2018). Aggarwal et al. (2017) used structure-guided methods to develop a lead molecule that targets the thioesterase activity of Pks13 (Figure 1B; Table 1). In another study, a series of thiophenes that kill *M*. *tuberculosis* were identified by targeting the N-terminal ACP_N_ domain of Pks13. Wilson et al. (2013) revealed that the compounds work by blocking the interaction of ACP_N_ with the FabD32 protein, which transfers the meromycolyl chain and is essential for the growth of mycobacteria. It can also inhibit the activity of PKs13 by blocking the TE domain of PKs13 (Ioerger et al., 2013; Zhang, W. et al., 2021). These results confirm Pks13 as a target for drugs against *M*. *tuberculosis* and highlight its potential in the development of new TB drugs that interfere with the critical pathway of mycolic acids synthesis. It further proves that structure-guided drug development is an effective method for producing new agents, although with limited application in the process of developing new antimycobacterial compounds.

### Enzymes related to mycolic acids transport

MmpL3 (Rv0206c) is an essential inner membrane protein in *M*. *tuberculosis* (Domenech et al., 2005). MmpL3 is responsible for the transport of mycolic acids in the form of trehalose monomycolate (TMM), the precursor of trehalose dimycolate (TDM) and mycolates bound to arabinogalactan that together forms the mycomembrane (Figure 1B). MmpL3 is critical for mycobacterial replication and viability (Belisle et al., 1997; Bhatt et al., 2005). MmpL3 has a periplasmic pore domain and a 12-helix transmembrane domain (Zhang et al., 2019), the structural data will greatly advance the development of MmpL3 inhibitors. Zhang et al. (2019) determined the crystal structure of the compound-MmpL3 and proved the direct interaction between the compound and MmpL3. Those compounds including the ethylenediamine derivative SQ109 (Table 1; Sacksteder et al., 2012; Tahlan et al., 2012; Li et al., 2014), indole-2-carboxamide ICA38 (Rao et al., 2013), adamantylurea AU1235 (Grzegorzewicz et al., 2012; McNeil et al., 2020). SQ109 is currently under Sequella’s US investigational new drug (IND) and completed three Phase 1 studies in the U.S. and two Phase 2 studies in drug-sensitive TB patients in Africa, in addition to the Phase 2b-3 study in Russia (Bukhdruker et al., 2020). SQ-109 appears to be safe and well-tolerated in human studies, with mild to moderate, dose-dependent gastrointestinal discomfort being the most frequently observed adverse events (Heinrich et al., 2015).

### Unknown target mycolic acids inhibitors

The new anti-TB drugs Delamanid (Table 1) producing nitric oxide, block the synthesis of mycolic acids, further damaging the stability of cell membranes (Matsumoto et al., 2006; Xavier and Lakshmanan, 2014). Preclinical studies showed that Delamanid is not genotoxic or potentially carcinogenic, and it has been approved for marketing by the State Food and Drug Administration in 2018 Clinical trials with a treatment plan containing Delamanid are underway in the United States because of the current increase in TB cases among AIDS patients (Nguyen et al., 2020). Pretomanid (PA-824), a bicyclic nitroimidazoles, is a novel anti-tuberculosis agent (Lenaerts et al., 2005). PA824 is a pro-drug that operates directly as a NO donor, it has many attractive characteristics as a potential TB therapy, most notably its novel mechanism of action. PA824 involves a dual-mode function, disrupting mycolic acids synthesis pathway for aerobic bacteria (Table 1; Tyagi et al., 2005; Manjunatha et al., 2009) and respiratory poisoning for anaerobic bacteria (Singh et al., 2008; Bahuguna and Rawat, 2020; Priyanka et al., 2020). PA-824 is converted by deazaflavin nitroreductase to nitrous oxide (NO) and other toxic products, that accumulate within bacteria and obstruct normal electron flow and homeostasis, which abrogate *M*. *tuberculosis* growth. The TB Alliance’s new drug application (NDA) for the novel TB drug candidate pretomanid has been accepted for review by the United States Food and Drug Administration (FDA). The application was submitted for the use of pretomanid as part of a new regimen, in combination with bedaquiline and linezolid, in the treatment of extensively drug-resistant (XDR) TB, treatment intolerant multidrug-resistant (MDR) TB, and treatment non-responsive MDR-TB (Conradie et al., 2020), the effectiveness of PA824 in treating MDR/XDR TB with combinations of first-line TB medications has been confirmed. In conclusion, the key enzymes in the mycolic acids synthesis pathway can represent an important approach in the discovery of anti-TB drugs.

## Biosynthesis and potential targets of arabinogalactan and lipoarabinomannan

LAM and AG are the two major mycobacterial cell wall (lipo) polysaccharides, which contain a structurally similar arabinan domain that is highly branched. AG is synthesized on a decaprenyl phosphate (C_50_-P) lipid carrier and then attached to peptidoglycan and mycolic acids (Jankute et al., 2012). The phosphatidyl-myo-inositol mannosidase (PIMs), lipomannan (LM) and LAM are essential for the survival and pathogenicity of *M*. *tuberculosis* (Torrelles et al., 2009); the enzymes involved in the biosynthesis of AG and LAM could be the ideal targets for anti-TB drug discovery.

### Arabinosyltransferase (EmbA/B/C, Rv3794/ Rv3795/Rv3793)

Arabinosyltransferase (Emb) is a key enzyme responsible for the biosynthesis of AG and LAM. EmbC is mainly responsible for the synthesis of LAM and associates with ethambutol (EMB) resistance (Zhang et al., 2020). Ethambutol competitively inhibits the substrate to bind EmbB and EmbC subunits. *M*. *tuberculosis* has difficulties in developing drug resistance without affecting its complex cell wall construction (Figure 1C). The above research enabled the optimization in the use of ethambutol and the development of new drugs targeting Emb proteins (Jankute et al., 2012; Tan et al., 2020a). Singh et al. (2019) analyzed the US Food and Drug Administration (FDA) library for EmbC, and selected drugs with higher binding affinity to EmbC, such as Terlipressin and Amikacin. The analysis of the size and shape of minimal energy landscape area shows greater stability of the EmbC-Terlipressin complex than the other drugs. It demonstrates the EmbC binding potential of the drug Terlipressin and Amikacin (Figure 1C), further underlining the urgency to develop new and improved treatments, with the reconsideration of existing drugs representing a potential route shortcut.

### Enzymes related to dTDP-L-rhamnose synthesis (RmlA/B/C/D)

The rhamnose-GlcNAc disaccharide (RmlA-D, Rv0334/Rv3464/Rv3465/Rv3266c) linker is fundamental for the structural integrity of the mycobacterial cell wall (Li et al., 2006; Qu et al., 2021). *M*. *tuberculosis* encode four RmlA-D enzymes involved in the synthesis of the donor dTDP-L-rhamnose beginning with dTTP and glucose-phosphate (Dhaked et al., 2019). Van et al., used a biological layer interference method to identify inhibitory lead compounds that bind to RmlB and RmlC and determined that Ri03 affects the viability of streptococcus and mycobacteria (Figure 1C). Thus, it can be used as a lead compound in the development of a new class of antibiotics against the biosynthesis of dTDP-L-rhamnose in pathogenic bacteria (van der Beek et al., 2019). Ravichandran et al. (2020) used the Super Natural-II database with AutoDock4.0 to perform a virtual screening of the RmlD protein and found a potential inhibitor of RmlD, thus inhibiting the synthesis of the cell wall in *M*. *tuberculosis*. The above studies prove that RmlB-D are potential new anti-TB drug targets, although no mature drug is available yet, and much research still needs to be performed.

### Key enzymes of arabinose donor (DprE1, DprE2)

DprE1 (Rv3790) and DprE2 (Rv3791) are key enzymes in the biosynthesis of arabinose donors (DPA; Figure 1C; Bhutani et al., 2015). Benzothiazolinones (BTZ) target DprE1, and BTZ043 (Table 1; phase II drug) is the most effective among these inhibitors, showing high potency (MIC of 1 ng/ml) against *M*. *tuberculosis* H37Rv, multidrug-resistant and extensively resistant *M*. *tuberculosis* isolates (Makarov et al., 2009; Trefzer et al., 2010; Zhang et al., 2018). PBTZ169 (Table 1; phase I drug) was optimized from BTZ043 by pharmacochemistry and exhibits an increased efficacy and safety (Makarov et al., 2014; Shi et al., 2018). Subsequently, many compounds with different scaffold structures inhibit enzymes in covalent or non-covalent way (Shirude et al., 2013). For example, TBA7371 (Azaindole derivative), which is a non-covalent inhibitor of DprE1 inhibitors, completed phase I clinical trials (Table 1; Chatterji et al., 2014; Zhang et al., 2018; Robertson et al., 2021; Figure 1C). Another phenotypic screening effort around the carbostyril core results in the discovery of OPC-167832. OPC-167832 in regimens combined with delamanid showed superior efficacy to a standard RHZE regimen (rifampicin + INH + pyrazinamide + ethambutol) in mice. However, trials need to compare the proportion of subjects with favorable outcomes in each experimental treatment arm with OPC-167832, delamanid and bedaquiline versus patients receiving a standard RHZE regimen (Table 1; Hariguchi et al., 2020). These results confirm DprE1 as a target for drug against *M*. *tuberculosis*, since it interferes with the critical pathway of mycolic acids synthesis.

### Arabinofuranosyltransferase (AftA, AftB, AftC, AftD)

The AftA (Rv3792), AftB (Rv3805c), and AftC (Rv2673) genes encode for the enzyme arabinofuranosyltransferase (ArafT), which is responsible for the polymerization of the arabinofuranyl (Araf) residues of DPA into the arabinose components AG and LAM (Figure 1D; Giri et al., 2019). Kolly et al. (2015) explored the transcription of the GtrA (Rv3789) protein and its adjacent genes and found that AftA prime the transfer of the first arabinose residue to the galactose chain. AftA is co-expressed with GtrA, DprE1 and DprE2. AftC in *M*. *tuberculosis* is an α-1,3 arabinosyltransferase involved in the branching of α-1,5 linear arabinan of both l LAM and AG (Zhang et al., 2011). AftD (Rv0236c) is the largest glycosyltransferase in the genome of *M*. *tuberculosis*. Glycan array analysis shows that AftD binds to complex arabinose glycans (Figure 1D; Tan et al., 2020b). All the identified arabinosyltransferases are essential in the growth of *M*. *tuberculosis* and are potential targets of new anti-TB drugs.

## Biosynthesis and potential targets of peptidoglycan

The biosynthesis of PG is a highly coordinated process composed of three stages of sequential reactions. In the first stage, UDP-GlcNAc is synthesized by the acetylation and uridylation of essential enzyme GlmU (Rv1018c) beginning with the fructose-1-phosphate (Figure 1E). In the second stage, the synthesis of UDP-MurNAc-pentapeptide is catalyzed by the MurA-F (Rv1315/ Rv0482/Rv2152c/Rv2155c/Rv2158c/Rv2157c) ligase pathway (Rani and Khan, 2016). Starting from UDP-GlcNAc, the enzyme MurA adds phosphoenol pyruvate to form UDP-enoylpyr uvyl-GlcNAc, which is in turn transformed in UDP-MurNAc upon reduction of the enoylpyruvyl moiety to a lactoyl ether moiety with NADPH as an electron hydrogen donor catalyzed by MurB (Figure 1E; Squeglia et al., 2018). Besides, the MurX (Rv2156c) transfers the nucleotide glycopentapeptide to the decallyl phosphate to form the first membrane-bound peptidoglycan precursor (lipid I; Siricilla et al., 2014). Subsequently, the β (1 → 4) bond is formed between GlcNAc of UDP-GlcNAc and MurNAc/Glyc of lipid I by the MurG (Rv2153c) enzyme, thus forming lipid II (Zhang, L. et al., 2021). An enzyme with transglycosylase and transpeptidase activity is necessary for the final stage of peptidoglycan synthesis (Raghavendra et al., 2018). Finally, the Lcp1 enzyme links the arabinogalactan complex to the peptidoglycan (Alderwick et al., 2015). Mur ligases enable the development of cell walls through the cytoplasmic to periplasmic biosynthesis (Sangshetti et al., 2017; Figure 1E). Thus, the Mur ligases may be an ideal target for the discovery of new antibiotics/anti-TB drugs.

### Targeting GlmU, MurA-F

GlmU is a bifunctional enzyme with glucosamine-1-phosphate acetyltransferase activity and N-acetylglucosamine-1-phosphate uridine transferase activity considered as a potential drug target (Agarwal et al., 2021). Kolly et al. (2015); Han et al. (2019) screened many compounds from different natural products against *M*. *tuberculosis* by DTNB colorimetry. The results showed that dicoumarin has a good inhibitory effect on GlmU acetyltransferase (Figure 1E). MurA-F represent important transferase/ligase in the steps of peptidoglycan biosynthesis. The amide ligases MurC, MurD, MurE and MurF are characterized by the same catalytic function because they possess similar amino acid regions and preserved comparable structural properties (Rani and Khan, 2016). Kumar et al. (2020) analyzed two peptide compound libraries (Asinex and ChemDiv) based on the structure of MurA, and they found that four compounds have acceptable pharmacokinetic properties. After screening FDA-approved drugs from two repositories, they found that sulfadoxine and pyrimethamine have stable interaction with MurB, while rifilast and sildenafil have the most reliable interaction with MurE (Figure 1E; Rani et al., 2020). D-cycloserine is a structural analog of D-alanine, it can competitively inhibit two essential enzymes in the synthesis of peptidoglycan: alanine racemase (Alr) and D-Ala: D-Ala ligase, which are involved in pentapeptide core formation (Figure 1E; Prosser and de Carvalho, 2013). Cycloserine is a broad-spectrum antibiotic introduced in 1952 and was recommended by the World Health Organization (WHO) to be administered to MDR-TB patients. Due to fewer patients with drug-resistant disease have been reported than for some other second-line antituberculosis drugs (Caminero et al., 2010; Yu, X. et al., 2018). A capbramycin analogram kills nonreplicating *M*. *tuberculosis* at low concentrations, with strong synergistic effects with SQ641 (a MurX inhibitor; Figure 1E; Siricilla et al., 2015).

### Targeting β-lactamase

β-lactam (Bla) antibiotics inhibit the transpeptidase activity of penicillin-binding proteins (PBPs) to block the cross-linking of peptidoglycans (Hugonnet and Blanchard, 2007). However, BlaC (Rv2068c, β-lactamase) in *M*. *tuberculosis* is a broad-spectrum hydrolase that renders ineffective the vast majority of relevant β-lactam compounds currently in use (Lu et al., 2020). Therefore, the development of inhibitors that bind and inhibit BlaC but cannot be hydrolyzed by BlaC is urgently needed. Some studies showed that meropenem in combination with clavulanic acid (a BlaC inhibitor) enhance its activity to effectively kill non-replicating *M*. *tuberculosis* (Hugonnet and Blanchard, 2007; Tiberi et al., 2016; van Rijn et al., 2019). Tolerability of intravenous meropenem with amoxicillin-clavulanate was poor at all phase II clinical trial doses, which maybe an obstacle of meropenem in second-line regimens (De Jager et al., 2022).

The structure of *M*. *tuberculosis* peptidoglycan is atypical in that it is mainly a 3 → 3 cross-link formed by the L,D-transpeptidase (Ldts; Lavollay et al., 2008; Gupta et al., 2010; Dubee et al., 2012). Penicillin and cephalosporin classes of β-lactams cannot inhibit L,D- transpeptidase function; however, carbapenems (eg. meropenem and imipenem) inactivate its function (Soroka et al., 2015; Lopez Quezada et al., 2020). Avibactam is another β-lactamase inhibitor based on a diazabicyclooctane (DBO) scaffold containing a cyclic urea rather than a β-lactam ring (Edoo et al., 2018). Ceftazidime was first marketed almost 40 years ago and has no activity against *M. tuberculosis*. However, Ceftazidime-avibactam has remarkable sterilizing effect at clinically achievable concentrations (Deshpande et al., 2017). Edoo et al. (2018) optimized the diazabicyclooctane (DBO) scaffold of avibactam (a β-lactamase inhibitor) and found that DBO 15a (DBO azide derivative) inhibited the LdtMt2 effectively. It was shown that optimization of avibactam to inhibit Ldt is an attractive strategy to obtain drugs with selective activity against Mycobacterium.

## Potential drugs in pre-clinical phase

Currently, several potential drugs are under discovery and in a pre-clinical phase, in addition to Delamanid and PA-824, which are in clinical phase III. Some of the drugs in pre-clinical studies targeting the synthesis of cell wall are JSF-3285, CPZEN-45, TAM-16, and Indolcarboxamides (NITD-304, NITD-349). These drugs target common components such as PKs-13, WecA, KasA, and MmpL3 (Table 1). The TB drug development pipeline is promising in researching new drugs targeting DprE1 such as BTZ-043, PBTZ-169, TBA7371, and SQ109 (Table 1). The safety, tolerability, and efficacy of these drugs on healthy individuals and TB patients is under evaluation.

## Conclusion

TB remains to be one of the leading causes of morbidity and mortality illness throughout the world. With the increasing number of multidrug-resistant and extensively drug-resistant TB worldwide, more effective drugs need to be developed to shorten the treatment time. However, the discovery of new drugs requires a better understanding of the virulence factors of *M*. *tuberculosis* in the host. The cell wall of *M*. *tuberculosis* plays an important role in the long-term infection and virulence of this pathogen. The biosynthetic pathways of the cell wall components reveal valuable drug targets (essential for the growth of *M*. *tuberculosis* and lack of their homologs in mammalian systems) that are the basis of present drugs and/or have the potential for new anti-TB drugs discovery.

## Author contributions

XX wrote the original draft. JZ reviewed and edited the manuscript with help from CG and LP. ZH and BD contributed the schematic diagram. JZ and CW provided the conceptualization and fundings. All authors contributed to the article and approved the submitted version.

## Funding

This work was supported by the National Natural Science Foundation of China (31400040), the Fundamental Research Funds for the Central Universities (YJ201985).

## Conflict of interest

The authors declare that the research was conducted in the absence of any commercial or financial relationships that could be construed as a potential conflict of interest.

## Publisher’s note

All claims expressed in this article are solely those of the authors and do not necessarily represent those of their affiliated organizations, or those of the publisher, the editors and the reviewers. Any product that may be evaluated in this article, or claim that may be made by its manufacturer, is not guaranteed or endorsed by the publisher.

## References

[ref1] Agarwal M. Soni V. Kumar S. Singha B. Nandicoori V. K. (2021). Unique C-terminal extension and interactome of mycobacterium tuberculosis GlmU impacts it’s in vivo function and the survival of pathogen. Biochem. J. 478, 2081–2099. doi: 10.1042/BCJ20210170, PMID: 33955473

[ref2] Aggarwal A. Parai M. K. Shetty N. Wallis D. Woolhiser L. Hastings C.. (2017). Development of a novel Lead that targets *M*. *tuberculosis* polyketide synthase 13. Cells 170, 249–259.e25. doi: 10.1016/j.cell.2017.06.025, PMID: 28669536 PMC5509550

[ref3] Alderwick L. J. Birch H. L. Mishra A. K. Eggeling L. Besra G. S. (2007). Structure, function and biosynthesis of the mycobacterium tuberculosis cell wall: arabinogalactan and lipoarabinomannan assembly with a view to discovering new drug targets. Biochem. Soc. Trans. 35, 1325–1328. doi: 10.1042/BST0351325 17956343

[ref4] Alderwick L. J. Harrison J. Lloyd G. S. Birch H. L. (2015). The mycobacterial Cell Wall--peptidoglycan and arabinogalactan. Cold Spring Harb. Perspect. Med. 5:a021113. doi: 10.1101/cshperspect.a021113, PMID: 25818664 PMC4526729

[ref5] Alfhili M. A. Hussein H. A. M. Park Y. Lee M. H. Akula S. M. (2021). Triclosan induces apoptosis in Burkitt lymphoma-derived BJAB cells through caspase and JNK/MAPK pathways. Apoptosis 26, 96–110. doi: 10.1007/s10495-020-01650-0, PMID: 33387145

[ref6] Asselineau J. Lederer E. (1950). Structure of the mycolic acids of mycobacteria. Nature 166, 782–783. 14780245 10.1038/166782a0

[ref7] Bahuguna A. Rawat D. S. (2020). An overview of new antitubercular drugs, drug candidates, and their targets. Med. Res. Rev. 40, 263–292. 31254295 10.1002/med.21602

[ref8] Belisle J. T. Vissa V. D. Sievert T. Takayama K. Brennan P. J. Besra G. S. (1997). Role of the major antigen of mycobacterium tuberculosis in cell wall biogenesis. Science 276, 1420–1422. doi: 10.1126/science.276.5317.1420, PMID: 9162010

[ref9] Bell L. C. K. Noursadeghi M. (2018). Pathogenesis of HIV-1 and mycobacterium tuberculosis co-infection. Nat. Rev. Microbiol. 16, 80–90. doi: 10.1038/nrmicro.2017.128 29109555

[ref10] Bhat Z. S. Rather M. A. Maqbool M. Lah H. U. L. Yousuf S. K. Ahmad Z. (2017). Cell wall: a versatile fountain of drug targets in mycobacterium tuberculosis. Biomed. Pharmacother. 95, 1520–1534. doi: 10.1016/j.biopha.2017.09.036, PMID: 28946393

[ref11] Bhatt A. Kremer L. Dai A. Z. Sacchettini J. C. Jacobs W. R. Jr. (2005). Conditional depletion of Kas A, a key enzyme of mycolic acid biosynthesis, leads to mycobacterial cell lysis. J Bacteriol. 187, 7596–7606. doi: 10.1128/JB.187.22.7596-7606.2005 16267284 PMC1280301

[ref12] Bhutani I. Loharch S. Gupta P. Madathil R. Parkesh R. (2015). Structure, dynamics, and interaction of mycobacterium tuberculosis (Mtb) Dpr E1 and Dpr E2 examined by molecular modeling, simulation, and electrostatic studies. PLoS One 10:e0119771. doi: 10.1371/journal.pone.0119771, PMID: 25789990 PMC4366402

[ref13] Bukhdruker S. Varaksa T. Grabovec I. Marin E. Shabunya P. Kadukova M.. (2020). Hydroxylation of Antitubercular drug candidate, SQ109, by mycobacterial cytochrome P450. Int. J. Mol. Sci. 21. doi: 10.3390/ijms21207683, PMID: 33081390 PMC7589583

[ref005] Burki T. (2019). BPaL approved for multidrug-resistant tuberculosis. Lancet Infect Dis. 19, 1063–1064. doi: 10.1016/S1473-3099(19)30489-X 31559963

[ref14] Caminero J. A. Sotgiu G. Zumla A. Migliori G. B. (2010). Best drug treatment for multidrug-resistant and extensively drug-resistant tuberculosis. Lancet Infect Dis. 10, 621–629. doi: 10.1016/S1473-3099(10)70139-0 20797644

[ref15] Chatterji M. Shandil R. Manjunatha M. R. Solapure S. Ramachandran V. Kumar N.. (2014). 1,4-azaindole, a potential drug candidate for treatment of tuberculosis. Antimicrob. Agents Chemother. 58, 5325–5331. doi: 10.1128/AAC.03233-14, PMID: 24957839 PMC4135869

[ref16] Choi K. H. Kremer L. Besra G. S. Rock C. O. (2000). Identification and substrate specificity of beta -ketoacyl (acyl carrier protein) synthase III (mtFabH) from mycobacterium tuberculosis. J. Biol. Chem. 275, 28201–28207. doi: 10.1074/jbc.M003241200, PMID: 10840036

[ref17] Conradie F. Diacon A. H. Ngubane N. Howell P. Everitt D. Crook A. M.. (2020). Treatment of highly drug-resistant pulmonary Tuberculosis. N. Engl. J. Med. 382, 893–902. doi: 10.1056/NEJMoa1901814 32130813 PMC6955640

[ref18] Daffe M. (2015). The cell envelope of tubercle bacilli. Tuberculosis 95, S155–S158. doi: 10.1016/j.tube.2015.02.024, PMID: 25819158

[ref19] De Jager V. Gupte N. Nunes S. Barnes G. L. van Wijk R. C. Mostert J.. (2022). Early bactericidal activity of meropenem plus clavulanate (with or without rifampin) for tuberculosis: The COMRADE randomized, phase 2A clinical trial. Am J. Respir. Crit. Care 205, 1228–1235. doi: 10.1164/rccm.202108-1976OC PMC9872811 35258443

[ref20] Delaine T. Bernardes-Génisson V. Quémard A. Constant P. Meunier B. Bernadou J. (2010). Development of isoniazid-NAD truncated adducts embedding a lipophilic fragment as potential bi-substrate InhA inhibitors and antimycobacterial agents. Eur. J. Med. Chem. 45, 4554–4561. doi: 10.1016/j.ejmech.2010.07.016, PMID: 20696503

[ref21] Deshpande D. Srivastava S. Chapagain M. Magombedze G. Martin K. R. Cirrincione K. N.. (2017). Ceftazidime-avibactam has potent sterilizing activity against highly drug-resistant tuberculosis. Sci. Adv. 3:e1701102. doi: 10.1126/sciadv.1701102, PMID: 28875168 PMC5576880

[ref22] Dhaked D. K. Bala Divya M. Guruprasad L. (2019). A structural and functional perspective on the enzymes of mycobacterium tuberculosis involved in the L-rhamnose biosynthesis pathway. Prog. Biophys. Mol. Biol. 145, 52–64. doi: 10.1016/j.pbiomolbio.2018.12.004, PMID: 30550737

[ref23] Domenech P. Reed M. B. Barry C. E. 3rd. (2005). Contribution of the Mycobacterium tuberculosis MmpL protein family to virulence and drug resistance. Infect Immun. 73, 3492–3501. doi: 10.1128/IAI.73.6.3492-3501.2005 15908378 PMC1111821

[ref24] Dubee V. Triboulet S. Mainardi J. L. Etheve-Quelquejeu M. Gutmann L. Marie A.. (2012). Inactivation of Mycobacterium tuberculosis l, d-transpeptidase LdtMt(1) by carbapenems and cephalosporins. Antimicrob Agents Chemother. 56, 4189–4195. doi: 10.1128/AAC.00665-12 22615283 PMC3421625

[ref25] Edoo Z. Iannazzo L. Compain F. de la Sierra L. Gallay I. van Tilbeurgh H.. (2018). Synthesis of avibactam derivatives and activity on beta-lactamases and peptidoglycan biosynthesis enzymes of Mycobacteria. Chemistry. 24, 8081–8086. doi: 10.1002/chem.201800923 29601108

[ref26] Farjallah A. Chiarelli L. R. Forbak M. Degiacomi G. Danel M. Goncalves F.. (2021). A coumarin-based analogue of thiacetazone as dual covalent inhibitor and potential fluorescent label of HadA in Mycobacterium tuberculosis. ACS Infect Dis. 7, 552–565. doi: 10.1021/acsfecdis.0c00325 33617235 PMC8022203

[ref27] Freundlich J. S. Wang F. Vilcheze C. Gulten G. Langley R. Schiehser G. A.. (2009). Triclosan derivatives: towards potent inhibitors of drug-sensitive and drug-resistant Mycobacterium tuberculosis. Chem. MedChem. 4, 241–248. doi: 10.1002/cmdc.200800261 PMC3541007 19130456

[ref28] Gavalda S. Léger M. van der Rest B. Stella A. Bardou F. Montrozier H.. (2009). The Pks13/FadD32 crosstalk for the biosynthesis of mycolic acids in mycobacterium tuberculosis. J. Biol. Chem. 284, 19255–19264. doi: 10.1074/jbc.M109.006940, PMID: 19436070 PMC2740550

[ref29] Giri A. Safi H. Cabibbe A. M. Gupta S. Narang A. Tyagi G.. (2019). Lack of association of novel mutation Asp397Gly in aftB gene with ethambutol resistance in clinical isolates of Mycobacterium tuberculosis. Tuberculosis (Edinb). 115, 49–55. doi: 10.1016/j.tube.2019.01.004 30948176

[ref30] Grzegorzewicz A. E. Pham H. Gundi V. A. K. B. Scherman M. S. North E. J. Hess T.. (2012). Inhibition of mycolic acid transport across the mycobacterium tuberculosis plasma membrane. Nat. Chem. Biol. 8, 334–341. doi: 10.1038/nchembio.794, PMID: 22344175 PMC3307863

[ref31] Guardia A. Gulten G. Fernandez R. Gómez J. Wang F. Convery M.. (2016). N-Benzyl-4-((heteroaryl)methyl)benzamides: a new class of direct NADH-dependent 2-trans Enoyl-acyl carrier protein reductase (InhA) inhibitors with Antitubercular activity. ChemMedChem 11, 687–701. doi: 10.1002/cmdc.201600020, PMID: 26934341

[ref32] Gupta R. Lavollay M. Mainardi J. L. Arthur M. Bishai W. R. Lamichhane G. (2010). The mycobacterium tuberculosis protein LdtMt2 is a nonclassical transpeptidase required for virulence and resistance to amoxicillin. Nat. Med. 16, 466–469. doi: 10.1038/nm.2120, PMID: 20305661 PMC2851841

[ref33] Han X. Chen C. Yan Q. Jia L. Taj A. Ma Y. (2019). Action of Dicumarol on Glucosamine-1-phosphate acetyltransferase of GlmU and mycobacterium tuberculosis. Front. Microbiol. 10:1799. doi: 10.3389/fmicb.2019.01799, PMID: 31481936 PMC6710349

[ref34] Hariguchi N. Chen X. Hayashi Y. Kawano Y. Fujiwara M. Matsuba M.. (2020). OPC-167832, a novel Carbostyril derivative with potent Antituberculosis activity as a DprE1 inhibitor. Antimicrob. Agents Chemother. 64:e02020-19. doi: 10.1128/AAC.02020-19, PMID: 32229496 PMC7269503

[ref35] He X. Alian A. Ortiz de Montellano P. R. (2007). Inhibition of the mycobacterium tuberculosis enoyl acyl carrier protein reductase InhA by arylamides. Bioorg. Med. Chem. 15, 6649–6658. doi: 10.1016/j.bmc.2007.08.013, PMID: 17723305 PMC2020492

[ref36] Heinrich N. Dawson R. du Bois J. Narunsky K. Horwith G. Phipps A. J.. (2015). Early phase evaluation of SQ109 alone and in combination with rifampicin in pulmonary TB patients. J. Antimicrob. Chemother. 70, 1558–1566. doi: 10.1093/jac/dku553, PMID: 25630641

[ref37] Hugonnet J. E. Blanchard J. S. (2007). Irreversible inhibition of the mycobacterium tuberculosis beta-lactamase by clavulanate. Biochemistry 46, 11998–12004. doi: 10.1021/bi701506h 17915954 PMC2593862

[ref38] Inoyama D. Awasthi D. Capodagli G. C. Tsotetsi K. Sukheja P. Zimmerman M.. (2020). A preclinical candidate targeting mycobacterium tuberculosis KasA. Cell. Chem. Biol. 27, 560–570.e10. doi: 10.1016/j.chembiol.2020.02.007 32197094 PMC7245553

[ref39] Ioerger T. R. O’Malley T. Liao R. Guinn K. M. Hickey M. J. Mohaideen N.. (2013). Identification of new drug targets and resistance mechanisms in mycobacterium tuberculosis. PLoS One 8:e75245. doi: 10.1371/journal.pone.0075245, PMID: 24086479 PMC3781026

[ref001] Ishizaki Y. Hayashi C. Inoue K. Igarashi M. Takahashi Y. Pujari V.. (2013). Inhibition of the first step in synthesis of the mycobacterial cell wall core, catalyzed by the GlcNAc-1-phosphate transferase WecA, by the novel caprazamycin derivative CPZEN-45. J. Biol. Chem. 288, 30309–30319. doi: 10.1074/jbc.M113.492173 23986448 PMC3798496

[ref40] Jankute M. Cox J. A. G. Harrison J. Besra G. S. (2015). Assembly of the mycobacterial Cell Wall. Annu. Rev. Microbiol. 69, 405–423. doi: 10.1146/annurev-micro-091014-104121 26488279

[ref41] Jankute M. Grover S. Rana A. K. Besra G. S. (2012). Arabinogalactan and lipoarabinomannan biosynthesis: structure, biogenesis and their potential as drug targets. Future Microbiol. 7, 129–147. doi: 10.2217/fmb.11.123, PMID: 22191451

[ref42] Kalscheuer R. Palacios A. Anso I. Cifuente J. Anguita J. Jacobs W. R. Jr.. (2019). The mycobacterium tuberculosis capsule: a cell structure with key implications in pathogenesis. Biochem. J. 476, 1995–2016. doi: 10.1042/BCJ20190324, PMID: 31320388 PMC6698057

[ref43] Kamsri P. Koohatammakun N. Srisupan A. Meewong P. Punkvang A. Saparpakorn P.. (2014). Rational design of InhA inhibitors in the class of diphenyl ether derivatives as potential anti-tubercular agents using molecular dynamics simulations. SAR QSAR Environ. Res. 25, 473–488. doi: 10.1080/1062936X.2014.898690, PMID: 24785640

[ref003] Kaur P. Potluri V. Ahuja V. K. Naveenkumar C. N. Krishnamurthy R. V. Gangadharaiah S. T.. (2021). A multi-targeting pre-clinical candidate against drug-resistant tuberculosis. Tuberculosis (Edinb). 129:102104. doi: 10.1016/j.tube.2021.102104 34214859

[ref44] Kolly G. S. Mukherjee R. Kilacsková E. Abriata L. A. Raccaud M. Blaško J.. (2015). GtrA protein Rv3789 is required for Arabinosylation of arabinogalactan in mycobacterium tuberculosis. J. Bacteriol. 197, 3686–3697. doi: 10.1128/JB.00628-15, PMID: 26369580 PMC4626892

[ref45] Kouassi A. F. Kone M. Keita M. Esmel A. Megnassan E. N’Guessan Y. T.. (2015). Computer-aided design of orally bioavailable pyrrolidine carboxamide inhibitors of enoyl-acyl carrier protein reductase of Mycobacterium tuberculosis with favorable pharmacokinetic profiles. Int. J. Mol. Sci. 16, 29744–29771. doi: 10.3390/ijms161226196 26703572 PMC4691139

[ref46] Kremer L. Douglas J. D. Baulard A. R. Morehouse C. Guy M. R. Alland D.. (2000). Thiolactomycin and related analogues as novel anti-mycobacterial agents targeting KasA and KasB condensing enzymes in mycobacterium tuberculosis. J. Biol. Chem. 275, 16857–16864. doi: 10.1074/jbc.M000569200, PMID: 10747933

[ref47] Kremer L. Nampoothiri K. M. Lesjean S. Dover L. G. Graham S. Betts J.. (2001). Biochemical characterization of acyl carrier protein (AcpM) and malonyl-CoA:AcpM transacylase (mtFabD), two major components of Mycobacterium tuberculosis fatty acid synthase II. J. Biol. Chem. 276, 27967–27974. doi: 10.1074/jbc.M103687200 11373295

[ref48] Kumar P. Saumya K. U. Giri R. (2020). Identification of peptidomimetic compounds as potential inhibitors against MurA enzyme of mycobacterium tuberculosis. J. Biomol. Struct. Dyn. 38, 4997–5013. doi: 10.1080/07391102.2019.1696231, PMID: 31755364

[ref49] Lai C. Y. Cronan J. E. (2003). Beta-ketoacyl-acyl carrier protein synthase III (FabH) is essential for bacterial fatty acid synthesis. J. Biol. Chem. 278, 51494–51503. doi: 10.1074/jbc.M308638200, PMID: 14523010

[ref50] Lavollay M. Arthur M. Fourgeaud M. Dubost L. Marie A. Veziris N.. (2008). The peptidoglycan of stationary-phase mycobacterium tuberculosis predominantly contains cross-links generated by L D-transpeptidation. J. Bacteriol. 190, 4360–4366. doi: 10.1128/JB.00239-08, PMID: 18408028 PMC2446752

[ref51] Lee W. Engels B. (2013). Clarification on the decarboxylation mechanism in KasA based on the protonation state of key residues in the acyl-enzyme state. J. Phys. Chem. B 117, 8095–8104. doi: 10.1021/jp403067m, PMID: 23768199

[ref52] Lenaerts A. J. Gruppo V. Marietta K. S. Johnson C. M. Driscoll D. K. Tompkins N. M.. (2005). Preclinical testing of the nitroimidazopyran PA-824 for activity against mycobacterium tuberculosis in a series of in vitro and in vivo models. Antimicrob. Agents Chemother. 49, 2294–2301. doi: 10.1128/AAC.49.6.2294-2301.2005, PMID: 15917524 PMC1140539

[ref53] Li W. Upadhyay A. Fontes F. L. North E. J. Wang Y. Crans D. C.. (2014). Novel insights into the mechanism of inhibition of MmpL3, a target of multiple pharmacophores in mycobacterium tuberculosis. Antimicrob. Agents Chemother. 58, 6413–6423. doi: 10.1128/AAC.03229-14, PMID: 25136022 PMC4249373

[ref54] Li W. Xin Y. McNeil M. R. Ma Y. (2006). rmlB and rmlC genes are essential for growth of mycobacteria. Biochem. Biophys. Res. Commun. 342, 170–178. doi: 10.1016/j.bbrc.2006.01.130 16472764

[ref004] Li W. Sanchez-Hidalgo A. Jones V. de Moura V. C. North E. J. Jackson M. (2017). Synergistic interactions of MmpL3 inhibitors with antitubercular compounds in vitro. Antimicrob Agents chemother. 61, e02399–e02316. doi: 10.1128/AAC.02399-16 28115355 PMC5365669

[ref55] Lopez Q. L. Smith R. Lupoli T. J. Edoo Z. Li X. Gold B.. (2020). Activity-based protein profiling reveals that cephalosporins selectively active on non-replicating Mycobacterium tuberculosis bind multiple protein families and spare peptidoglycan transpeptidases. Front Microbiol. 11:1248. doi: 10.3389/fmicb.2020.01248 32655524 PMC7324553

[ref56] Lu Z. Wang H. Zhang A. Liu X. Zhou W. Yang C.. (2020). Structures of Mycobacterium tuberculosis penicillin-binding protein 3 in complex with five beta-lactam antibiotics reveal mechanism of inactivation. Mol. Pharmacol. 97, 287–294. doi: 10.1124/mol.119.118042 32086254

[ref57] Makarov V. Lechartier B. Zhang M. Neres J. Sar A. M. Raadsen S. A.. (2014). Towards a new combination therapy for tuberculosis with next generation benzothiazinones. EMBO Mol. Med. 6, 372–383. doi: 10.1002/emmm.201303575, PMID: 24500695 PMC3958311

[ref58] Makarov V. Manina G. Mikusova K. Möllmann U. Ryabova O. Saint-Joanis B.. (2009). Benzothiazinones kill mycobacterium tuberculosis by blocking arabinan synthesis. Science 324, 801–804. doi: 10.1126/science.1171583, PMID: 19299584 PMC3128490

[ref59] Manjunatha U. Boshoff H. I. M. Barry C. E. (2009). The mechanism of action of PA-824: novel insights from transcriptional profiling. Commun. Integr. Biol. 2, 215–218. doi: 10.4161/cib.2.3.7926, PMID: 19641733 PMC2717523

[ref60] Manjunatha U. H. SP S. R. Kondreddi R. R. Noble C. G. Camacho L. R. Tan B. H.. (2015). Direct inhibitors of InhA are active against Mycobacterium tuberculosis. Sci. Transl. Med. 7:269ra263. doi: 10.1126/scitranslmed.3010597 PMC4383039 25568071

[ref61] Matsumoto M. Hashizume H. Tomishige T. Kawasaki M. Tsubouchi H. Sasaki H.. (2006). OPC-67683, a nitro-dihydro-imidazooxazole derivative with promising action against tuberculosis in vitro and in mice. PLoS Med. 3:e466. doi: 10.1371/journal.pmed.0030466 17132069 PMC1664607

[ref62] McNeil M. B. O’Malley T. Dennison D. Shelton C. D. Sunde B. Parish T. (2020). Multiple mutations in Mycobacterium tuberculosis MmpL3 increase resistance to MmpL3 inhibitors. mSphere. 5, e00985–e00920. doi: 10.1128/mSphere.00985-20 33055263 PMC7565900

[ref63] Miotto P. Zhang Y. Cirillo D. M. Yam W. C. (2018). Drug resistance mechanisms and drug susceptibility testing for tuberculosis. Respirology. 23, 1098–1113. doi: 10.1111/resp.13393 30189463

[ref64] Nguyen T. V. A. Anthony R. M. Cao T. T. H. Bañuls A. L. Nguyen V. A. T. Vu D. H.. (2020). Delamanid resistance: update and clinical management. Clin. Infect. Dis. 71, 3252–3259. doi: 10.1093/cid/ciaa755, PMID: 32521000

[ref65] Oh T. J. Daniel J. Kim H. J. Sirakova T. D. Kolattukudy P. E. (2006). Identification and characterization of Rv3281 as a novel subunit of a biotin-dependent acyl-CoA carboxylase in mycobacterium tuberculosis H37Rv. J. Biol. Chem. 281, 3899–3908. doi: 10.1074/jbc.M511761200, PMID: 16354663 PMC1523427

[ref66] Ouyang F. Tang N. Zhang H. J. Wang X. Zhao S. Wang W.. (2018). Maternal urinary triclosan level, gestational diabetes mellitus and birth weight in Chinese women. Sci. Total Environ. 626, 451–457. doi: 10.1016/j.scitotenv.2018.01.102, PMID: 29353787 PMC5849787

[ref67] Parish T. Roberts G. Laval F. Schaeffer M. Daffé M. Duncan K. (2007). Functional complementation of the essential gene fabG1 of mycobacterium tuberculosis by mycobacterium smegmatis fabG but not Escherichia coli fabG. J. Bacteriol. 189, 3721–3728. doi: 10.1128/JB.01740-06, PMID: 17337570 PMC1913321

[ref68] Portevin D. de Sousa-D’Auria C. Houssin C. Grimaldi C. Chami M. Daffé M.. (2004). A polyketide synthase catalyzes the last condensation step of mycolic acid biosynthesis in mycobacteria and related organisms. Proc. Natl. Acad. Sci. U. S. A. 101, 314–319. doi: 10.1073/pnas.0305439101, PMID: 14695899 PMC314182

[ref69] Portevin D. de Sousa-D’Auria C. Montrozier H. Houssin C. Stella A. Lanéelle M. A.. (2005). The acyl-AMP ligase FadD32 and AccD4-containing acyl-CoA carboxylase are required for the synthesis of mycolic acids and essential for mycobacterial growth: identification of the carboxylation product and determination of the acyl-CoA carboxylase components. J. Biol. Chem. 280, 8862–8874. doi: 10.1074/jbc.M408578200, PMID: 15632194

[ref70] Prasad M. S. Bhole R. P. Khedekar P. B. Chikhale R. V. (2021). Mycobacterium enoyl acyl carrier protein reductase (InhA): a key target for antitubercular drug discovery. Bioorg. Chem. 115:105242. doi: 10.1016/j.bioorg.2021.105242, PMID: 34392175

[ref71] Priyanka T. A. Maske P. Mote C. Dighe V. (2020). Gestational and lactational exposure to triclosan causes impaired fertility of F1 male offspring and developmental defects in F2 generation. Environ. Pollut. 257:113617. doi: 10.1016/j.envpol.2019.113617 31780364

[ref72] Prosser G. A. de Carvalho L. P. (2013). Kinetic mechanism and inhibition of mycobacterium tuberculosis D-alanine:D-alanine ligase by the antibiotic D-cycloserine. FEBS J. 280, 1150–1166. doi: 10.1111/febs.12108, PMID: 23286234

[ref73] Qu D. Zhao X. Sun Y. Wu F. L. Tao S. C. (2021). Mycobacterium tuberculosis thymidylyltransferase RmlA is negatively regulated by Ser/Thr protein kinase PknB. Front Microbiol. 12:643951. doi: 10.3389/fmicb.2021.643951 33868202 PMC8044546

[ref74] Raghavendra T. Patil S. Mukherjee R. (2018). Peptidoglycan in mycobacteria: chemistry, biology and intervention. Glycoconj. J. 35, 421–432. doi: 10.1007/s10719-018-9842-7, PMID: 30232572

[ref75] Rani C. Khan I. A. (2016). UDP-GlcNAc pathway: potential target for inhibitor discovery against *M*. *tuberculosis*. Eur. J. Pharm. Sci. 83, 62–70. doi: 10.1016/j.ejps.2015.12.013 26690048

[ref76] Rani J. Silla Y. Borah K. Ramachandran S. Bajpai U. (2020). Repurposing of FDA-approved drugs to target MurB and MurE enzymes in Mycobacterium tuberculosis. J. Biomol. Struct Dyn. 38, 2521–2532. doi: 10.1080/07391102.2019.1637280 31244382

[ref77] Rao S. P. Lakshminarayana S. B. Kondreddi R. R. Herve M. Camacho L. R. Bifani P.. (2013). Indolcarboxamide is a preclinical candidate for treating multidrug-resistant tuberculosis. Sci. Transl. Med. 5:214ra168. doi: 10.1126/scitranslmed.3007355 24307692

[ref78] Ravichandran R. Ridzwan N. F. W. Mohamad S. B. (2020). Ensemble-based high-throughput virtual screening of natural ligands using the Super Natural-II database against cell-wall protein dTDP-4-dehydrorhamnose reductase (RmlD) in Mycobacterium tuberculosis. J. Biomol. Struct Dyn. 40, 5069–5078. doi: 10.1080/07391102.2020.1867641 33382017

[ref79] Robertson G. T. Ramey M. E. Massoudi L. M. Carter C. L. Zimmerman M. Kaya F.. (2021). Comparative analysis of pharmacodynamics in the C3HeB/FeJ mouse tuberculosis model for DprE1 inhibitors TBA-7371, PBTZ169, and OPC-167832. Antimicrob. Agents Chemother. 65:e0058321. doi: 10.1128/AAC.00583-21, PMID: 34370580 PMC8522729

[ref80] Sacco E. Covarrubias A. S. O’Hare H. M. Carroll P. Eynard N. Jones T. A.. (2007). The missing piece of the type II fatty acid synthase system from mycobacterium tuberculosis. Proc. Natl. Acad. Sci. U. S. A. 104, 14628–14633. doi: 10.1073/pnas.0704132104, PMID: 17804795 PMC1976197

[ref81] Sacksteder K. A. Protopopova M. Barry C. E. Andries K. Nacy C. A. (2012). Discovery and development of SQ109: a new antitubercular drug with a novel mechanism of action. Future Microbiol. 7, 823–837. doi: 10.2217/fmb.12.56, PMID: 22827305 PMC3480206

[ref002] Salomon J. J. Galeron P. Schulte N. Morow P. R. Severynse-Stevens D. Huwer H.. (2013). Biopharmaceutical in vitro characterization of CPZEN-45, a drug candidate for inhalation therapy of tuberculosis. Ther Deliv. 4, 915–923. doi: 10.4155/tde.13.62 23919471

[ref82] Sangshetti J. N. Joshi S. S. Patil R. H. Moloney M. G. Shinde D. B. (2017). Mur ligase inhibitors as anti-bacterials: a comprehensive review. Curr. Pharm Des. 23, 3164–3196. doi: 10.2174/1381612823666170214115048 28201974

[ref83] Shi J. Lu J. Wen S. Zong Z. Huo F. Luo J.. (2018). *In vitro* activity of PBTZ169 against multiple mycobacterium species, Antimicrob agents Chemother, vol. 62. doi: 10.1128/AAC.01314-18 PMC6201125 30150479

[ref84] Shirude P. S. Shandil R. Sadler C. Naik M. Hosagrahara V. Hameed S.. (2013). Azaindoles: noncovalent DprE1 inhibitors from scaffold morphing efforts, kill mycobacterium tuberculosis and are efficacious *in vivo*. J. Med. Chem. 56, 9701–9708. doi: 10.1021/jm401382v, PMID: 24215368

[ref85] Singh R. Manjunatha U. Boshoff H. I. M. Ha Y. H. Niyomrattanakit P. Ledwidge R.. (2008). PA-824 kills non-replicating mycobacterium tuberculosis by intracellular NO release. Science 322, 1392–1395. doi: 10.1126/science.1164571, PMID: 19039139 PMC2723733

[ref86] Singh A. Somvanshi P. Grover A. (2019). Drug repurposing against arabinosyl transferase (EmbC) of mycobacterium tuberculosis: essential dynamics and free energy minima based binding mechanics analysis. Gene 693, 114–126. doi: 10.1016/j.gene.2019.01.029, PMID: 30716439

[ref87] Siricilla S. Mitachi K. Skorupinska-Tudek K. Swiezewska E. Kurosu M. (2014). Biosynthesis of a water-soluble lipid I analogue and a convenient assay for translocase I. Anal. Biochem. 461, 36–45. doi: 10.1016/j.ab.2014.05.018, PMID: 24939461 PMC4296562

[ref88] Siricilla S. Mitachi K. Wan B. Franzblau S. G. Kurosu M. (2015). Discovery of a capuramycin analog that kills nonreplicating mycobacterium tuberculosis and its synergistic effects with translocase I inhibitors. J. Antibiot. (Tokyo) 68, 271–278. doi: 10.1038/ja.2014.133, PMID: 25269459 PMC4382465

[ref89] Slama N. Jamet S. Frigui W. Pawlik A. Bottai D. Laval F.. (2016). The changes in mycolic acid structures caused by hadC mutation have a dramatic effect on the virulence of Mycobacterium tuberculosis. Mol Microbiol. 99, 794–807. doi: 10.1111/mmi.13266 26538472

[ref90] Soroka D. Li de la Sierra-Gallay I. Dubée V. Triboulet S. van Tilbeurgh H. Compain F.. (2015). Hydrolysis of clavulanate by mycobacterium tuberculosis beta-lactamase BlaC harboring a canonical SDN motif. Antimicrob. Agents Chemother. 59, 5714–5720. doi: 10.1128/AAC.00598-15, PMID: 26149997 PMC4538473

[ref91] Squeglia F. Ruggiero A. Berisio R. (2018). Chemistry of peptidoglycan in mycobacterium tuberculosis life cycle: an off-the-wall balance of synthesis and degradation. Chemistry 24, 2533–2546. doi: 10.1002/chem.201702973, PMID: 28925518

[ref92] Tadolini M. Garcia-Garcia J. M. Blanc F. X. Borisov S. Goletti D. Motta I.. (2020). On tuberculosis and COVID-19 co-infection. Eur. Respir J. 56:2002328. doi: 10.1183/13993003.02328-2020 32586888 PMC7315815

[ref93] Tahlan K. Wilson R. Kastrinsky D. B. Arora K. Nair V. Fischer E.. (2012). SQ109 targets MmpL3, a membrane transporter of trehalose monomycolate involved in mycolic acid donation to the cell wall core of mycobacterium tuberculosis. Antimicrob. Agents Chemother. 56, 1797–1809. doi: 10.1128/AAC.05708-11, PMID: 22252828 PMC3318387

[ref94] Tan Y. Z. Rodrigues J. Keener J. E. Zheng R. B. Brunton R. Kloss B.. (2020a). Cryo-EM structure of arabinosyltransferase EmbB from mycobacterium smegmatis. Nat. Commun. 11:3396. doi: 10.1038/s41467-020-17202-8, PMID: 32636380 PMC7341804

[ref95] Tan Y. Z. Zhang L. Rodrigues J. Zheng R. B. Giacometti S. I. Rosário A. L.. (2020b). Cryo-EM structures and regulation of Arabinofuranosyltransferase AftD from mycobacteria. Mol. Cell 78, 683–699.e11. doi: 10.1016/j.molcel.2020.04.014 32386575 PMC7263364

[ref96] Tiberi S. Sotgiu G. D’Ambrosio L. Centis R. Abdo Arbex M. Alarcon Arrascue E.. (2016). Comparison of effectiveness and safety of imipenem/clavulanate- versus meropenem/clavulanate-containing regimens in the treatment of MDR- and XDR-TB. Eur. Respir. J. 47, 1758–1766. doi: 10.1183/13993003.00214-2016, PMID: 27076583

[ref97] Torrelles J. B. DesJardin L. E. MacNeil J. Kaufman T. M. Kutzbach B. Knaup R.. (2009). Inactivation of mycobacterium tuberculosis mannosyltransferase pimB reduces the cell wall lipoarabinomannan and lipomannan content and increases the rate of bacterial-induced human macrophage cell death. Glycobiology 19, 743–755. doi: 10.1093/glycob/cwp042, PMID: 19318518 PMC2688391

[ref98] Trefzer C. Rengifo-Gonzalez M. Hinner M. J. Schneider P. Makarov V. Cole S. T.. (2010). Benzothiazinones: prodrugs that covalently modify the decaprenylphosphoryl-beta-D-ribose 2′-epimerase DprE1 of mycobacterium tuberculosis. J. Am. Chem. Soc. 132, 13663–13665. doi: 10.1021/ja106357w, PMID: 20828197

[ref99] Tyagi S. Nuermberger E. Yoshimatsu T. Williams K. Rosenthal I. Lounis N.. (2005). Bactericidal activity of the nitroimidazopyran PA-824 in a murine model of tuberculosis. Antimicrob. Agents Chemother. 49, 2289–2293. doi: 10.1128/AAC.49.6.2289-2293.2005, PMID: 15917523 PMC1140529

[ref100] van der Beek S. L. Zorzoli A. Çanak E. Chapman R. N. Lucas K. Meyer B. H.. (2019). Streptococcal dTDP-L-rhamnose biosynthesis enzymes: functional characterization and lead compound identification. Mol. Microbiol. 111, 951–964. doi: 10.1111/mmi.14197, PMID: 30600561 PMC6487966

[ref101] van Rijn S. P. Zuur M. A. Anthony R. Wilffert B. van Altena R. Akkerman O. W.. (2019). Evaluation of carbapenems for treatment of multi- and extensively drug-resistant Mycobacterium tuberculosis. Antimicrob Agents Chemother. 63, e01489–e01418. doi: 10.1128/AAC.01489-18 30455232 PMC6355583

[ref102] Vilcheze C. Jacobs W. R. Jr. (2014). Resistance to isoniazid and Ethionamide in mycobacterium tuberculosis: genes, mutations, and causalities. Microbiol. Spectr. 2:MGM2-0014-2013. doi: 10.1128/microbiolspec.MGM2-0014-2013 PMC6636829 26104204

[ref103] Vilcheze C. Jacobs W. R. Jr. (2019). The isoniazid paradigm of killing, resistance, and persistence in mycobacterium tuberculosis. J. Mol. Biol. 431, 3450–3461. doi: 10.1016/j.jmb.2019.02.016, PMID: 30797860 PMC6703971

[ref104] Vilcheze C. Molle V. Carrere-Kremer S. Leiba J. Mourey L. Shenai S.. (2014). Phosphorylation of KasB regulates virulence and acid-fastness in Mycobacterium tuberculosis. PLoS Pathog. 10:e1004115. doi: 10.1371/journal.ppat.1004115 24809459 PMC4014462

[ref105] Vosatka R. Kratky M. Vinsova J. (2018). Triclosan and its derivatives as antimycobacterial active agents. Eur. J. Pharm Sci. 114, 318–331. doi: 10.1016/j.ejps.2017.12.013 29277667

[ref106] Wall M. D. Oshin M. Chung G. A. C. Parkhouse T. Gore A. Herreros E.. (2007). Evaluation of N-(phenylmethyl)-4-[5-(phenylmethyl)-4,5,6,7-tetrahydro-1H-imidazo[4,5-c]pyridin- 4-yl]benzamide inhibitors of mycobacterium tuberculosis growth. Bioorg. Med. Chem. Lett. 17, 2740–2744. doi: 10.1016/j.bmcl.2007.02.078, PMID: 17418567

[ref107] Wilson R. Kumar P. Parashar V. Vilchèze C. Veyron-Churlet R. Freundlich J. S.. (2013). *Antituberculosis thiophenes* define a requirement for Pks13 in mycolic acid biosynthesis. Nat. Chem. Biol. 9, 499–506. doi: 10.1038/nchembio.1277, PMID: 23770708 PMC3720791

[ref108] World Health, O. (2021) Global tuberculosis report 2021. Geneva: World Health Organization. Licence: CC BY-NY-SA 3.0 IGO

[ref109] Xavier A. S. Lakshmanan M. (2014). Delamanid: a new armor in combating drug-resistant tuberculosis. J. Pharmacol. Pharmacother. 5, 222–224. doi: 10.4103/0976-500X.136121, PMID: 25210407 PMC4156838

[ref006] Xu J. Li S. Y. Almeida D. V. Tasneen R. Barnes-Boyle K. Converse P. J.. (2019). Contribution of pretomanid to novel regimens containing bedaquiline with either linezolid or moxifloxacin and pyrazinamide in murine models of tuberculosis. Antimicrob Agents Chemother. 63, e00021–e00019. doi: 10.1128/AAC.00021-19 30833432 PMC6496099

[ref110] Yu M. Dou C. Gu Y. Cheng W. (2018). Crystallization and structure analysis of the core motif of the Pks13 acyltransferase domain from Mycobacterium tuberculosis. PeerJ. 6:e4728. doi: 10.7717/peerj.4728 29761048 PMC5944431

[ref111] Yu X. Zeng X. Shi W. Hu Y. Nie W. Chu N.. (2018). Validation of cycloserine efficacy in treatment of multidrug-resistant and extensively drug-resistant tuberculosis in Beijing. China. Antimicrob Agents Chemother. 62, e01824–e01817. doi: 10.1128/AAC.01824-17 29311073 PMC5826102

[ref112] Zhang J. Angala S. K. Pramanik P. K. Li K. Crick D. C. Liav A.. (2011). Reconstitution of functional mycobacterial arabinosyltransferase AftC proteoliposome and assessment of decaprenylphosphorylarabinose analogues as arabinofuranosyl donors. ACS Chem. Biol. 6, 819–828. doi: 10.1021/cb200091m, PMID: 21595486 PMC3158817

[ref113] Zhang W. Liu L. L. Lun S. Wang S. S. Xiao S. Gunosewoyo H.. (2021). Design and synthesis of mycobacterial pks13 inhibitors: Conformationally rigid tetracyclic molecules. Eur. J. Med. Chem. 213:113202. doi: 10.1016/j.ejmech.2021.113202, PMID: 33516983 PMC8689393

[ref114] Zhang L. Zhao Y. Gao Y. Wu L. Gao R. Zhang Q.. (2020). Structures of cell wall arabinosyltransferases with the anti-tuberculosis drug ethambutol. Science 368, 1211–1219. doi: 10.1126/science.aba9102, PMID: 32327601

[ref115] Zhang G. Guo S. Cui H. Qi J. (2018). Virtual screening of small molecular inhibitors against DprE1. Molecules. 23:524. doi: 10.3390/molecules23030524 29495447 PMC6017230

[ref116] Zhang B. Li J. Yang X. Wu L. Zhang J. Yang Y.. (2019). Crystal structures of membrane transporter MmpL3, an anti-TB drug target. Cell. 176:e613. doi: 10.1016/j.cell.2019.01.003 30682372

[ref117] Zhang L. Ramijan K. Carrion V. J. van der Aart L. T. Willemse J. van Wezel G. P.. (2021). An alternative and conserved cell wall enzyme that can substitute for the lipid II synthase MurG. mBio. 12, e03381–e03320. doi: 10.1128/mBio.03381-20 33824209 PMC8092295

